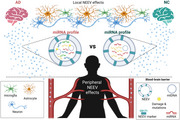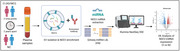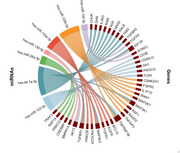# Biomarker potential of Neuronal Enriched Extracellular Vesicle miRNAs in Alzheimer’s Disease with multiethnic comparison

**DOI:** 10.1002/alz70861_108439

**Published:** 2025-12-23

**Authors:** Kumudu Subasinghe, Courtney Hall, Megan Rowe, Zhengyang Zhou, Robert Barber, Nicole Phillips

**Affiliations:** ^1^ University of North Texas Health Science Center, Fort Worth, TX USA; ^2^ Johns Hopkins University, Baltimore, MD USA

## Abstract

**Background:**

Alzheimer's Disease (AD) is a complex neurological illness influenced by genetics, epigenetics, and environmental factors. It is the primary cause of Dementia. Mexican Americans (MAs) are disproportionately affected by AD, leading to earlier onset, rapid cognitive decline compared to Non‐Hispanic Whites (NHWs). Early detection of cognitive impairment (CI) related to AD is crucial for effective treatment, as it allows for timely interventions that could slow disease progression and improve the quality of life for both patients and caregivers. Blood biomarkers are non‐invasive and cost‐effective approach for early AD detection. Extracellular vesicles (EVs) have emerged as promising blood biomarkers due to their ability of extending the early changes in the AD brain to the periphery through their biological cargo. We hypothesize that Neuronal‐enriched extracellular vesicle (NEEV) miRNA profiles differ between CI and healthy individuals, and that these profiles may contain specific miRNAs with differential expression linked to disease progression and relevant comorbidities (e.g., Type 2 diabetes, hyperlipidemia, hypertension), varying across different population groups.

**Method:**

We analyzed longitudinal plasma samples (collected at two time points, two years apart) from MAs and NHWs provided by the Texas Alzheimer's Research and Care Consortium (TARCC). The samples were processed using a two‐step method involving precipitation of total exosomes, followed by the capture of NEEVs with a biotinylated antibody targeting the neuronal surface marker CD171. miRNAs isolated from NEEVs were sequenced using next‐generation sequencing, and the data was analyzed for differential miRNA expression between CI and healthy controls over time using miRDeep2/DEseq2, QIAGEN RNA‐seq portal and Differential expression for repeated measures (DREAM).

**Result:**

We identified significant differential expression of hsa‐miR‐122‐5p in the CI group in both MAs and NHWs. Furthermore, we found population‐specific candidates with potential as biomarkers for AD precision medicine. Other population specific miRNAs identified may have biomarker potential in AD precision medicine. The miRNA target genes reflect their AD and related disease association and the key covariable effect on miRNA expression were identified through statistical analysis.

**Conclusion:**

Our study identifies several NEEV‐miRNA candidates with early blood biomarker potential for AD, providing insights through a multiethnic comparison.